# Microbial Community Structure and Associations During a Marine Dinoflagellate Bloom

**DOI:** 10.3389/fmicb.2018.01201

**Published:** 2018-06-06

**Authors:** Jin Zhou, Mindy L. Richlen, Taylor R. Sehein, David M. Kulis, Donald M. Anderson, Zhonghua Cai

**Affiliations:** ^1^Shenzhen Public Platform for Screening and Application of Marine Microbial Resources, Graduate School at Shenzhen, Tsinghua University, Shenzhen, China; ^2^Department of Biology, Woods Hole Oceanographic Institution, Woods Hole, MA, United States

**Keywords:** microbial community, algal bloom, dynamic process, network interaction, ecological function

## Abstract

Interactions between microorganisms and algae during bloom events significantly impacts their physiology, alters ambient chemistry, and shapes ecosystem diversity. The potential role these interactions have in bloom development and decline are also of particular interest given the ecosystem impacts of algal blooms. We hypothesized that microbial community structure and succession is linked to specific bloom stages, and reflects complex interactions among taxa comprising the phycosphere environment. This investigation used pyrosequencing and correlation approaches to assess patterns and associations among bacteria, archaea, and microeukaryotes during a spring bloom of the dinoflagellate *Alexandrium catenella*. Within the bacterial community, *Gammaproteobacteria* and *Bacteroidetes* were predominant during the initial bloom stage, while *Alphaproteobacteria, Cyanobacteria*, and *Actinobacteria* were the most abundant taxa present during bloom onset and termination. In the archaea biosphere, methanogenic members were present during the early bloom period while the majority of species identified in the late bloom stage were ammonia-oxidizing archaea and *Halobacteriales*. Dinoflagellates were the major eukaryotic group present during most stages of the bloom, whereas a mixed assemblage comprising diatoms, green-algae, rotifera, and other microzooplankton were present during bloom termination. Temperature and salinity were key environmental factors associated with changes in bacterial and archaeal community structure, respectively, whereas inorganic nitrogen and inorganic phosphate were associated with eukaryotic variation. The relative contribution of environmental parameters measured during the bloom to variability among samples was 35.3%. Interaction analysis showed that Maxillopoda, Spirotrichea, Dinoflagellata, and *Halobacteria* were keystone taxa within the positive-correlation network, while *Halobacteria*, Dictyochophyceae, Mamiellophyceae, and *Gammaproteobacteria* were the main contributors to the negative-correlation network. The positive and negative relationships were the primary drivers of mutualist and competitive interactions that impacted algal bloom fate, respectively. Functional predictions showed that blooms enhance microbial carbohydrate and energy metabolism, and alter the sulfur cycle. Our results suggest that microbial community structure is strongly linked to bloom progression, although specific drivers of community interactions and responses are not well understood. The importance of considering biotic interactions (e.g., competition, symbiosis, and predation) when investigating the link between microbial ecological behavior and an algal bloom’s trajectory is also highlighted.

## Introduction

Phytoplankton are a fundamental component of the marine ecosystem, playing multiple roles in matter cycling and the support of global biological and geochemical processes (Lindh et al., 2013). Under certain conditions, phytoplankton can be a double-edged sword, ecologically speaking. On the one hand, phytoplankton account for less than 1% of overall photosynthetic biomass on Earth, yet contribute at least half of the world’s net oxygen production (Field et al., 1998; Monier et al., 2015). On the other hand, some phytoplankton taxa form harmful algal blooms (HABs), which can adversely impact ecosystems and human health. For example, *Alexandrium* is a cosmopolitan dinoflagellate genus that proliferates annually worldwide, often forming dense blooms in near-shore waters. Blooms of certain toxin-producing species in these genus are responsible for widespread and significant impacts, including animal mortality events, and can contaminate shellfish with toxins that threaten human health (Bagatini et al., 2014). Estimates of the economic costs of these blooms exceed $20 million USD (Anderson et al., 2012), largely due to losses suffered by tourism and aquaculture industries.

The increase in the frequency and global distribution of HABs in recent decades has prompted research to identify the ecological and physiological factors that trigger the initiation and influence the magnitude of these events. Abiotic factors influencing HAB occurrence and dynamics include hydrodynamic processes, environmental conditions, and nutrient availability (Bouchouicha Smida et al., 2012; Lewitus et al., 2012), whereas biotic factors include grazing, pathogenicity, and parasitism (Paerl and Otten, 2013; Carnicer et al., 2015). Among the array of biotic drivers, microbial community composition (particularly bacteria) is increasingly cited as influencing HAB development (Teeling et al., 2012, 2016; Klindworth et al., 2014; Needham and Fuhrman, 2016). This is due in part to the role microbial communities play in mediating biogeochemical cycling, micro-food web structure, and production of essential elements that stimulate algal growth (Doucette, 1995; Ferrier et al., 2002; Segev et al., 2016), as well as absorbing essential elements (Adachi et al., 2003), exhibiting algicidal activity (Demuez et al., 2015; Bloh et al., 2016), inhibiting sexual reproduction (Sanders, 2014), and regulating algae-bacteria signaling (such as quorum sensing) (Zhou et al., 2016b). Viruses also play a critical role in the regulation of phytoplankton biomass, including host infection and lysis (Mizumoto et al., 2008; Ory et al., 2010). Nagasaki (2008) summarized the ecological relationships between dinoflagellates and diatoms, and their viruses, and concluded that viral infection is a significant factor driving population dynamics of algae.

Bacterial community structure during phytoplankton blooms is complex and changes during bloom progression depending on the algal species, physiological status, environmental conditions, and bloom stage (Zheng, 2011). Several studies have reported changes in bacterial community structure during natural and semi-natural (mesocosm) phytoplankton blooms (Riemann et al., 2000; Jones et al., 2010; Theroux et al., 2012), and certain dominant bacteria groups (e.g., *Roseobacter* and *Flavobacterium*) have been associated with blooms (Buchan et al., 2014). The structure and metabolic properties of these communities influence their ecological functions, which can include nutrient provision, release of organic compounds, and even as a competitor with algae for a particular ecological niche (Amin et al., 2015). Together, these behaviors create a regulatory network that operates throughout bloom formation, duration, and collapse (Tan et al., 2015; Needham and Fuhrman, 2016).

Previous studies have largely focused on bacteria taxa, and to our knowledge none have examined the wider spectrum of the microbial biosphere (i.e., archaea and microeukaryotes) over the complete bloom cycle, i.e., from bloom initiation through termination (Kodama et al., 2006; Ramanan et al., 2016). In addition, despite a relatively mature understanding of microbial dynamics during HAB events, identifying potential microbial relationships remains a significant challenge (Fuhrman et al., 2015). Only a few studies have examined network interactions during algal blooms with respect to inter- and intra-species patterns (Yang et al., 2017), thus limiting our current understanding of the complex interactions between microbes and algae. To contribute herefore, it is necessary to study bacterioplankton dynamics and their relationships (positive or negative) during phytoplankton blooms to further our understanding of algal-bacteria interactions.

This investigation used high throughput sequencing and association networking methods to analyze prokaryotic and eukaryotic microbial community dynamics during a natural dinoflagellate bloom to better characterize patterns of interaction and related ecological behavior during HAB events. We hypothesized that microbial community structure and succession is linked to specific bloom stages, thus driving complex interactions among taxa comprising the phycosphere environment. The specific aims of this study were to explore the population dynamics of microorganisms during an algal bloom, and to identify and characterize correlations among microbial taxa using network analysis. The ultimate goal of this study was to advance our current understanding of community structure and associations among microbial populations during algal blooms, and provide insights into the mechanisms underlying these interactions and dynamics.

## Materials and Methods

### Study Site, Sampling, and Environmental Parameters

Field studies were carried out in Salt Pond in the Nauset Marsh System on Cape Cod (MA, United States). Salt Pond is a small kettle pond (∼82,200 m^2^ in area) located in Eastham, MA (41°32′35 N, 70°37′40 W). The pond is connected to the Nauset Estuary by a shallow and narrow tidal channel approximately 1–3 m in depth, which permits tidal exchange with the wider marsh system (Crespo et al., 2011; Richlen et al., 2012). Average and maximum depths in the pond are ∼3.4 and 9 m, respectively (Crespo et al., 2011). Freshwater enters Salt Pond solely through groundwater or precipitation, as there are no stream or river inputs. The flushing rate measured via dye tracers is about 0.4–0.7 day^-1^ (Anderson and Stolzenbach, 1985) and groundwater discharge to the pond has been estimated using radioisotope data (radon) and salinity at ∼3200–4500 m^3^ day^-1^ (Crusius et al., 2005). Blooms of the dinoflagellate *Alexandrium catenella* recur annually during the spring, and originate from the germination of resting cysts found within the pond (Crespo et al., 2011; Ralston et al., 2014). The physical features of this system provide a natural mesocosm for the study of algal blooms, thus providing an ideal study site to track microbial dynamics over the course of a bloom cycle.

**FIGURE 1:**
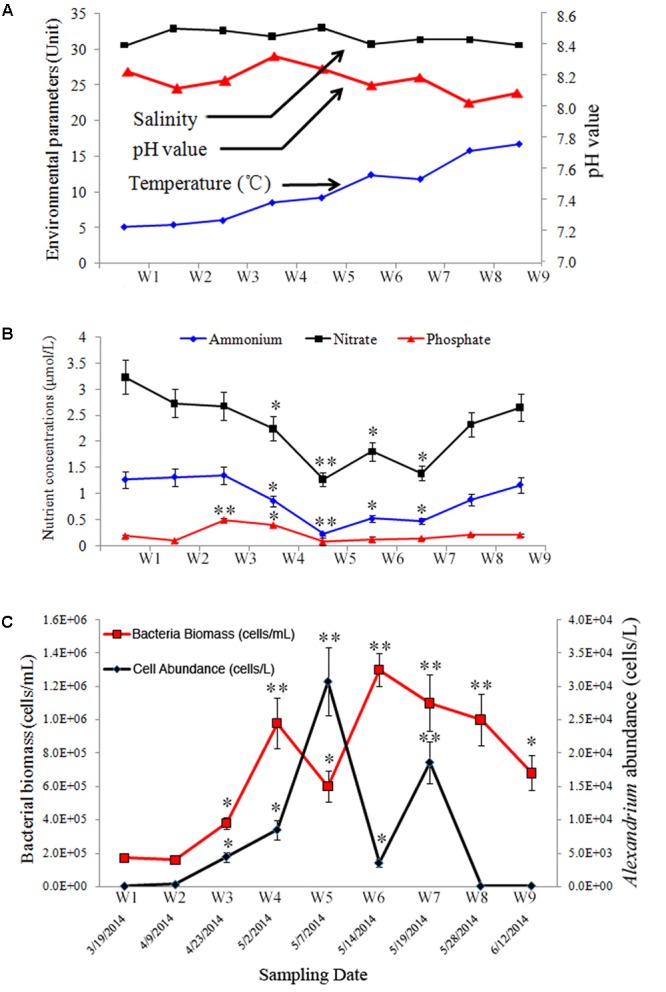
Environmental and biological parameters during a bloom of *Alexandrium catenella* in Salt Pond, Nauset Marsh, MA, United States. **(A)** temperature, salinity, and pH; **(B)** nutrients concentrations (nitrate [NO_3_^-^], ammonium [NH_4_^+^], and phosphate [PO_4_^3-^]); **(C)** abundances of *A. catenella* (black line) and total bacterioplankton (red line) in water samples collected over the course of the *A. catenella* bloom. Data points represent the mean + SD of triplicate measurements. The single asterisk (^∗^) and double asterisk (^∗∗^) indicated the significant difference compared with the first time-point (W1) at *P* < 0.05 and *P* < 0.01 levels, respectively.

Sampling was carried out in Salt Pond weekly from 19 March 2014 until 12 June 2014 (nine sampling time points; named W1 to W9; **Figure 1**). Water samples were collected in triplicate (2 L each) using a Niskin bottle from depths of about 0.5–1.0 m. Samples were first pre-filtered using a 300-mesh sieve, and were then collected on isopore filters with a diameter of 47 mm and pore size of 0.22 μm (Millipore, Burlington, MA, United States). Filters were stored at -80°C until analysis. A 50 mL fraction of each sample was preserved in 1% glutaraldehyde for enumerating total bacterial abundance. In addition, 500 mL of each sample was preserved with 1% Lugol’s iodine for phytoplankton identification and enumeration. This latter sample was further concentrated via settling and siphoning to produce a 25 mL concentrate. Algae were observed and counted under an optical microscope (×100 magnification).

During each sampling event, physicochemical parameters of the seawater were recorded. *In situ* measurements of salinity, pH, and temperature (°C) were collected using an YSI Professional Pro Meter (YSI Inc., Yellow Springs, OH, United States). The detection range of salinity and precision were 1–60 and 0.1, respectively. Ammonium (NH_4_^+^), nitrate (NO_3_^-^), and phosphate (PO_4_^3-^) were measured according to methods outlined below. NH_4_^+^ was measured according to the indophenol blue method using a spectrophotometer (DR/2800, Hach) (Hashihama et al., 2015). NO_3_^-^ analyses followed protocols from the Center for Microbial Oceanography at the University of Hawaii^1^. Phosphorus was measured by spectrophotometry following the formation of phosphomolybdic acid according to Murphy and Riley (1962). Nutrient analyses were performed on a Seal Analytical Continuous-Flow AutoAnalyzer 3 (United Kingdom). The detection limits of the aforementioned nutrient parameters were 0.05, 0.05, and 0.01 μmol/L, respectively. Approximate inorganic nitrogen/inorganic phosphate (IN/IP) values were measured according to methods of Murphy and Riley (1962) and Greenberg et al. (1992). Standards for NO_3_^-^, NO_2_^-^, and PO_4_^3-^ were obtained from Merck KGaA Biochemical Co., Ltd (Germany), and the purity was ≥99%.

### Bacterial Abundance

Seawater samples preserved in glutaraldehyde were stained with 4′-6′-diamidino-2-phenylindole (DAPI, Sigma, final concentration 1 μg/mL) for 15 min (Poter and Feig, 1980) and filtered onto 0.22 μm pore size polycarbonate filters (Millipore, Burlington, MA, United States). Bacterial cells were enumerated using an epifluorescent microscope (×1000 magnification). At least 10 fields of view and >50 cells were counted in each sample.

### DNA Extraction and PCR Amplification

DNA was extracted using a Fast DNA Spin Kit (Bio101, QbioGene, United States) according to the manufacturer’s instructions, and eluted in 50 μL TE buffer. DNA quality was estimated by the ratio of OD_260_/OD_280_ to verify that the value was >1.8 (NanoDrop^TM^ 2000), and stored at -20°C until further use.

Prokaryote 16S rRNA genes (V4–V5 hypervariable region) were amplified as described by Fierer et al. (2008) using the primers F515 (5′-GTGCCAGCMGCCGCGG-3′) and R907 (5′-CCGTCAATTCMTTTRAGTTT-3′). These primers were demonstrated *in silico* to target nearly all archaea and bacteria (Bates et al., 2011), and provide sufficient resolution for the taxonomic classification of microbial sequences (Liu et al., 2007). An 8-bp barcode was added to the forward primer, and samples were amplified in triplicate as described by Fierer et al. (2008). Replicate PCRs for each sample were subsequently pooled and purified using a QIAquick Gel Extraction Kit (Qiagen, Hilden, Germany).

Amplification of eukaryotes was performed using primers 547F (5′-CCAGCASCYGCGGTAATTCC-3′) and 952R (5′-ACTTTCGTTCTTGATYRA-3′), which target the V4 region of the 18S RNA gene. Amplifications were carried out in triplicate 50 μL PCRs comprising 0.5 μM of each primer, 1× GoTaq Flexi Reaction Buffer (Promega, Madison, WI, United States), 2.5 mM MgCl_2_, 200 mM dNTPs, 0.8 μg/mL BSA, 2.5 U of GoTaq Flexi DNA Polymerase (Promega), and 10 ng of total DNA. PCR cycling conditions were as follows: 2 min at 94°C; 10 cycles 94°C for 30 s, 65°C for 30 s (decreasing 1°C per cycle for 30 s, and 2°C for 30 s); 18 cycles of 94°C for 30 s, 55°C for 30 s, and 72°C for 30 s; and a final extension of 72°C for 10 min.

The PCR products were pooled, cleaned, and concentrated using a QIAquick PCR Purification Kit (Qiagen). A single composite sample for pyrosequencing was prepared by combining approximately equimolar amounts of PCR products from each sample. Sequencing was carried out by Biolinker Biotech. Co. Ltd. (Shanghai, China).

### Processing of 16S/18S Sequences

Raw sequences were processed and checked using Mothur and QIIMe software packages (Schloss et al., 2009). Sequencing data were denoised using the commands “shhh.flows” (translation of PyroNoise algorithm; Quince et al., 2009) and “pre.cluster” (Huse et al., 2010) on the Mothur platform. Chimeric sequences were identified and removed using UCHIME with the *de novo* method (Edgar et al., 2011). The raw sequence reads were trimmed and filtered according to a previous method (Huse et al., 2007). After low-quality reads were removed, representative sequences were annotated using an alignment tool (BLAST) against the Ribosomal Database Project and the Silva database augmented with 16S and 18S rRNA sequences from major marine taxa. We assigned operational taxonomic units (OTUs) at a 3% gene identity threshold using the UPARSE (version 7.1)^2^ pipeline (Edgar, 2013). The sequence data reported here have been deposited in the NCBI GenBank database (accession number SRP114998).

### Network Analysis

Interaction networks and co-occurrence patterns were examined according to Faust et al. (2012) and Yang et al. (2016). OTUs with average relative abundance of less than 0.1% were removed the microbial sequences, and a Spearman rank correlation matrix was created using the remaining data. Co-occurrence patterns were determined to be robust if the Spearman’s rank correlation coefficient (rho) was >0.6 and *P* < 0.05. These two filtering steps were applied to remove poorly represented OTUs and reduce network complexity, which assisted in determining the core microbial community in samples. An affiliation network was generated using the R package igraph, version 1.0.1. False positive correlations were filtered according to methods by Lima-Mendez et al. (2015) and Yambartsev et al. (2016). A network was constructed with edges consisting of correlations within an arbitrary *P*-value threshold. Unexpected links (a score below the thresholds) were identified, counted, and removed from the network. The false positives were discarded using the dirmult package in R. An affiliation network is defined as a network in which the members are affiliated with one another based on co-membership of a group or co-participation in some type of event. Direct connections among the different genera were extracted from the two-mode affiliation network using igraph’s bipartite.projection function. The nodes in the reconstructed network represented variable taxa, and the edges connecting the nodes represented correlations between genera. The topographic features of the network, including centrality and edge weights, were also analyzed using functions available in the igraph package. In addition, subsets of representative specialist genera from the samples were selected to help identify potential keystone taxa and their interactions within the network. Sub-networks were then generated from the meta-community networks using subgraph functions in the igraph package. Information on the target network was further organized in matrices and visualized in chord diagrams using the R package circlize, version 0.3.5.^3^

### Statistical Analyses

The α- and β-diversity indices for each sample were calculated using Mothur (Schloss et al., 2009). Statistically significant differences in taxa abundance were identified using Welch’s *t*-test in the STAMP program (Parks et al., 2014). The structure of the bacterial/archaeal/microeukaryotal community in each sample was compared using the 2STAGE analysis in the PRIMER (v6, PRIMER-E Ltd, Lutton, United Kingdom) (Clarke and Gorley, 2006). A heatmap of microbial communities was created using the PHYLOTEMP tool^4^ developed by Polson (2007), whereby relative abundance data are clustered based on the Bray–Curtis similarity algorithm. Principal component analysis (PCA) was used to display and compare microbial community composition among different samples, and canonical correspondence analysis (CCA) was used to link variations in microbial communities to environmental properties. Both analyses were carried out using Canoco 5 software. A variation partitioning analysis (VPA) was conducted to examine the contribution of environmental factors in influencing microbial community structure as determined by CCA analysis. Significant differences were defined as a *P* < 0.05 or *P* < 0.01. Functional analysis was performed in which bacterial gene functions were predicted from 16S rRNA gene-based microbial compositions using the PICRUSt algorithm to make inferences from KEGG annotated databases (Langille et al., 2013). The predicted metabolic functions of microbial communities at different algal bloom stages were identified using the PICRUSt (v1.0.0) protocol. For the environmental parameters, differences in various data were determined using analysis of variance (ANOVA) at the *P* < 0.05 significance level. Data analyses were performed using the SPSS software package 13.0 (Armonk, NY, United States).

## Results

### Environmental Parameters and Bloom Characteristics

Five primary stages were documented over the course of the bloom: pre- [W1], onset [W2–W3], exponential growth [W4], peak [W5], and decline/termination stages [W6–W9]. Over the duration of the sampling period, temperature, salinity, and pH values ranged from 5.2 to 16.8°C, 29.8 to 33.1, and 8.06 to 8.36, respectively (**Figure 1A**). Nutrient concentrations at each time point are shown in **Figure 1B**. NH_4_^+^+ NO_3_^-^ and PO_4_^3-^ ranged from 1.52 to 4.53 μmol/L and 0.12 to 0.51 μmol/L, respectively. Highest and lowest concentrations of NH_4_^+^ and NO_3_^-^ were detected during bloom onset and peak bloom stages, respectively (*P* < 0.01). A different trend was observed in PO_4_^3-^ concentrations in which PO_4_^3-^ was higher earlier in the bloom, and lower concentrations occurred during the peak- and decline bloom stages (*P* < 0.01). The approximate IN/IP ratio in surface waters ranged from 12.67 to 37.75.

*Alexandrium catenella* cell densities ranged from 1.5 × 10^2^ to 3.1 × 10^4^ cells/L over the sampling period (**Figure 1C**). The cellular morphology and size of *A. catenella* are shown in **Supplementary Figure S1**. Total abundance of bacteria ranged from 1.7 × 10^5^ to 1.2 × 10^6^ cells/mL, and the highest value appeared immediately after the bloom’s peak (**Figure 1C**) (*P* < 0.01). Micrographs of DAPI-stained filter samples are shown in **Supplementary Figure S2**. Total bacterial abundance was correlated with the *A. catenella* bloom, and a hysteresis phenomenon was observed in the population dynamics of the two groups.

### Biodiversity

**FIGURE 2:**
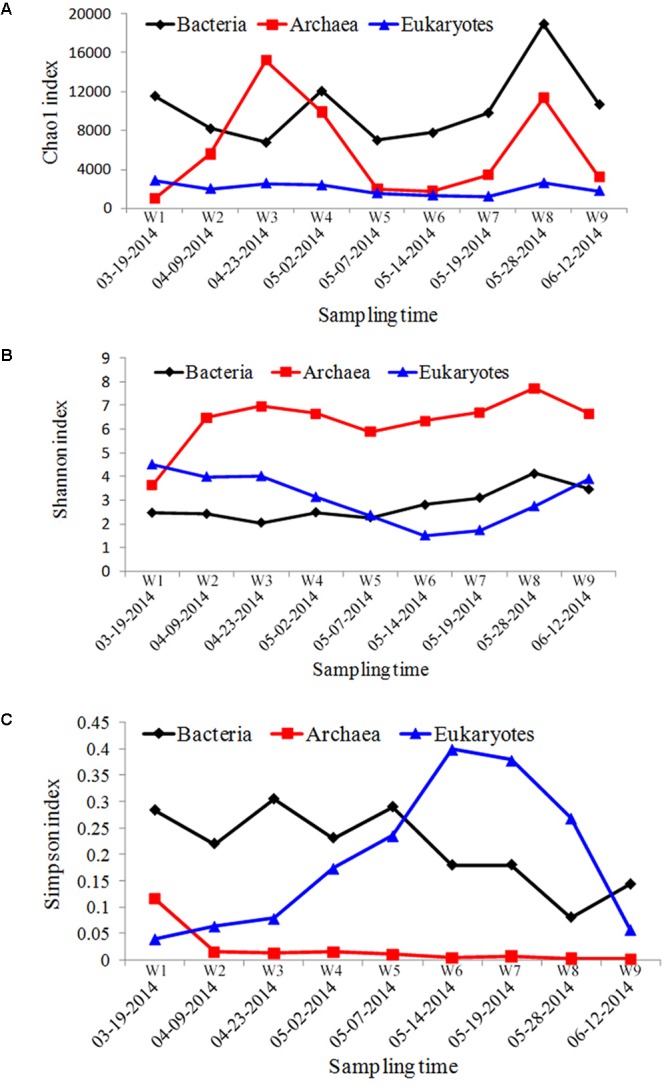
The α-diversity indices of microbial communities sampled from Salt Pond. **(A)** Chao1 index, **(B)** Shannon index, and **(C)** Simpson index.

Using a 97% similarity cut-off, a total of 4,859 bacterial OTUs, 517 archaea OTUs, and 3452 eukaryotes OTUs were obtained from the field samples. To investigate changes in biodiversity, stage variations in Chao1, Shannon, and Simpson indices were calculated for the bacterioplankton communities (**Figure 2A**). The Chao1 index calculated for the bacterial group remained relatively constant throughout the *A. catenella* bloom, and only increased at the starting point of bloom termination. The Shannon index was relatively constant throughout the bloom; Simpson indices exhibited a similar trend, but decreased slightly over the course of the bloom.

In the archaeal biosphere, fluctuations were observed throughout the entire sampling period. The highest Chao1 diversity index value appeared during bloom onset, and the lowest value was observed at the bloom’s peak (**Figure 2B**). The Shannon index values increased during the bloom, starting at the second time point (W2, 04-09-2014), whereas Simpson index values decreased from this time point.

Chao1 indices calculated for eukaryotic communities were significantly lower compared with those calculated for bacterial/archaeal communities, and were relatively stable throughout the bloom. Fluctuations were observed, however, in the Shannon and Simpson indices calculated for the different sampling periods. The most obvious change appeared at beginning of the bloom’s decline (W6, 05-14-2014), in which the lowest Shannon and highest Simpson diversity indices were observed (**Figure 2C**).

**FIGURE 3:**
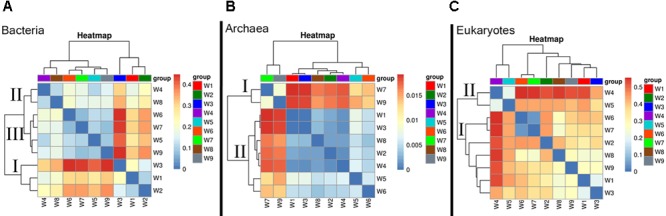
Heatmap illustrating Bray–Curtis similarities (β-diversity) based on taxonomic assignments (genus level). **(A)** Bacteria, **(B)** Archaea, and **(C)** Eukaryotes.

Patterns of microbial community beta diversity (among-sample differences in OTU composition) are shown in **Figure 3**, in which the color intensity reflects similarity in species composition. In the bacteria, the general dissimilarity trend among sampling periods was group I (W1–W3) > group II (W4 and W8) > group III (W5–W7 and W9). The biggest difference in community composition across samples was found for W3, which was collected during bloom development (**Figure 3A**). Archaea samples clustered into three groups that largely corresponded to the time period sampled (e.g., early, mid, late bloom), in spite of some overlap. The biggest difference among sampling periods was found at the decline stage, which had a comparatively higher beta-diversity indices (W7 and W9) *(group I)*; while the remaining samples exhibited similar community composition (group II) (**Figure 3B**). High similarity in eukaryotic species composition was found in a grouping comprised of samples from the early and late bloom stages (W1–W3 and W6–W9), whereas some differences appeared in samples collected during exponential growth (W4–W5). In this group, the highest beta-diversity index value was found at the peak stage of the bloom (**Figure 3C**).

### Microbial Community Dynamics

Bacterial community structure (phylum level) during the bloom is shown in **Supplementary Figure S3A**. *Bacteroidetes* and *Proteobacteria* were the two major dominant phyla during all bloom stages. The mean proportion of *Bacteroidetes* during the bloom’s peak (65.3%) was higher than during pre-bloom (30.6–51.5%) and post-bloom stages (21.5–53.1%). *Proteobacteria* exhibited an inverse pattern compared with *Bacteroidetes;* specifically, the *Gammaproteobacteria* dominated during the pre-bloom stage, while *Alphaproteobacteria* were predominant during the peak and decline stages (**Supplementary Figure S3B**). *Betaproteobacteria* and *Deltaproteobacteria* appeared during the post-bloom stage, and the proportional abundance of these groups was 5.1 and 2.8%, respectively. As the bloom progressed, significant changes in the bacterial community were observed. The *Cyanobacteria* cluster significantly increased (30%) during the onset of bloom decline (W6) and gradually decreased thereafter. Similarly, the *Actinobacteria* group gradually increased over time, and the maximum proportional abundance was observed during bloom decline (about 4.2–10.7%). Low proportions of *Spirochaetae*, Candidate division WS3, and *Acidobacteria* occupied <1% at different bloom phases (**Supplementary Figure S3A**).

**FIGURE 4:**
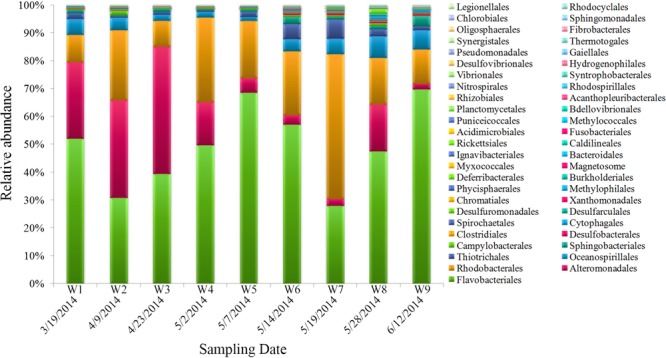
Bacterial community composition of samples (order level).

The dominant bacterial clades (top 10, order level) in samples were *Flavobacteriales, Alteromonadales, Rhodobacterales, Oceanospirillales, Thiotrichales, Sphingobacteriales, Campylobacterales, Desulfobacterales, Clostridiales*, and *Cytophagales*, which contributed >98% of the total quality reads (**Figure 4**). Among them, some taxa displayed obvious variations during algal bloom development. *Flavobacteriales* gradually increased during the bloom’s onset, reached maximum proportional abundance during the peak stage, and then declined along with the bloom; interestingly, abundance subsequently increased concurrent with the bloom’s termination. Similar to *Flavobacteriales, Rhodobacterales* increased significantly during the early bloom stages, reached highest proportional abundance when the bloom began to decline (W7), and decreased subsequently. *Alteromonadales* was the third most proportionally abundant order, and showed an overall decline as the bloom progressed. *Thiotrichales* and *Oceanospirillales* exhibited gradually increasing and decreasing patterns with bloom progression, respectively.

**FIGURE 5:**
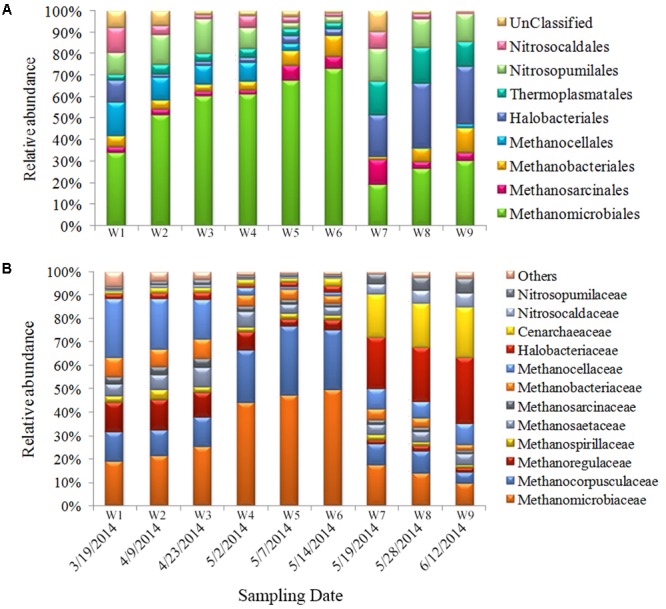
Archaeal community composition in samples. Relative abundances of the dominant groups at the order level **(A)** and family level **(B)**.

Compared with bacteria, relatively fewer archaea were detected. The ratio of archaea was about 6–10% of the total prokaryotic community. The sequences obtained in this study primarily fall within the Marine Groups I (*Thaumarchaeota*) and II (*Euryarchaeota*) (**Figure 5A**). Among the most abundant OTUs (genus level), >50% belonged to methanogenic species (*Methanomicrobiaceae, Methanocorpusculaceae, Methanoregullaceae*, and *Methanocellaceae*); 11–21% belonged to ammonia-oxidizing archaea (AOA) (*Nitrosocaldaceae* and *Nitrosocaldaceae*); 5–30% belonged to *Halobacteriaceae*; 3–10% belonged to *Thermoplasmatales*; and 2–9% belonged to other species (**Figure 5B**). The *Methanomicrobiales* were dominant during the first sampling period, and their proportional abundance increased significantly (to 60%) from bloom onset to exponential growth stages. *Halobacteriales* and *Thermoplasmatales* were present during bloom decline. The proportional abundance of these taxa increased during the late bloom stage and comprised 35–40% of the total archaeal community (**Figure 5A**).

**FIGURE 6:**
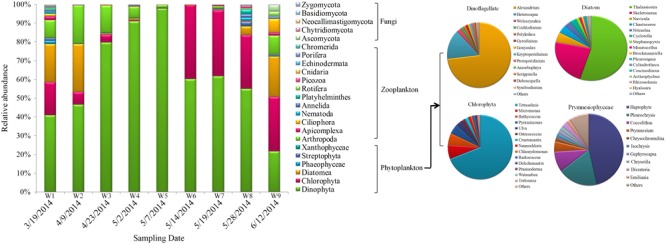
Eukaryotic community composition in samples. Relative abundances of the dominant groups at the phylum level. The right panel shows the composition of primary phytoplankton groups, including Dinoflagellata, Chlorophyta, diatoms, and Prymnesiophyceae.

Operational taxonomic units profiles of eukaryotic community structure during algal blooms in Salt Pond are shown in **Figure 6**. Dinophyta was clearly the major dominant phyla throughout the sampling period. During the pre-bloom stage, Chlorophyta, Diatomea, and Arthropoda species comprised the majority of the eukaryotic community. Over time, dinoflagellates (especially *Alexandrium* sp. and *Heterocapsa* sp.) significantly increased and reached peak abundance (>90%) at the height of the bloom (W5, 5/07/2014). During the later bloom stages (W7–W9), dinoflagellates were gradually replaced by other algal taxa, including Chlorophyta (mainly *Tetraselmis* sp. and *Micromonas* sp.), Diatomea (*Thalassiosira* sp. and *Skeletonema* sp.), and other low-abundance algae (*Prymnesiophyceae, Phaeophyceae*, and *Streptophyta*). In addition to the phytoplankton, zooplankton also exhibited distinct dynamics during the bloom. During the pre-bloom stage, Arthropoda, Apicomplexa, and some taxa (Nematoda, Annelida, Platyhelminthes, and Ascomycota) co-occurred. During the bloom’s peak stage, their abundances were significantly decreased and most of 18S OTUs were occupied by *Alexandrium* sp. When the algal bloom entered the termination stages, eukaryotic diversity gradually increased, and multiple organisms (algae, ciliate and fungi) co-occurred during this bloom phase (**Figure 6**).

### Cluster Analysis

**FIGURE 7:**
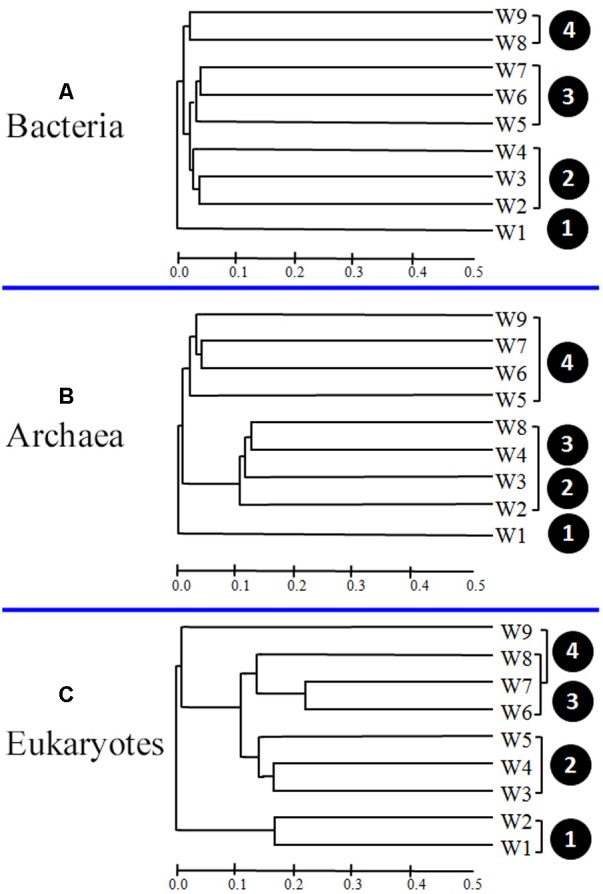
Bray–Curtis dissimilarity-based dendrogram at the operational taxonomic unit (OTU) level illustrating groups (**A**, Bacteria; **B**, Archaea; and **C**, Eukaryotes) in samples.

To determine whether the apparent differences in microbial community composition among samples significantly correlated with the algal bloom stage, cluster analyses were carried out. As shown in **Figure 7A**, the bacterial samples were divided into four clusters according to the different bloom stages (pre- [W1], onset and exponential growth [W2–W4], peak and initial decline [W5–W7], and termination stages [W8 and W9]).

Differences in archaeal community composition among bloom stages were also depicted by assemblage analysis (**Figure 7B**). Samples from the pre-bloom stage (W1) were clustered together and separated from samples collected during bloom onset (W2–W4). Interestingly, a few samples (W8 and W9) exhibited some similarities with the samples collected during exponential growth and decline bloom stages (W5–W7).

Analysis of 18S OTU profiles also revealed obvious temporal structuring. W1 and W2 formed the first group (pre-bloom and initiation of bloom onset), W3–W5 formed the second group (onset and exponential growth stages), and third group comprised the remaining samples (W6–W9), collected during the bloom decline stages. The distribution showed a distinct temporal progression, with overlap at some phases (**Figure 7C**).

### Correlation of Microbial Community Structure With Environmental Factors

**FIGURE 8:**
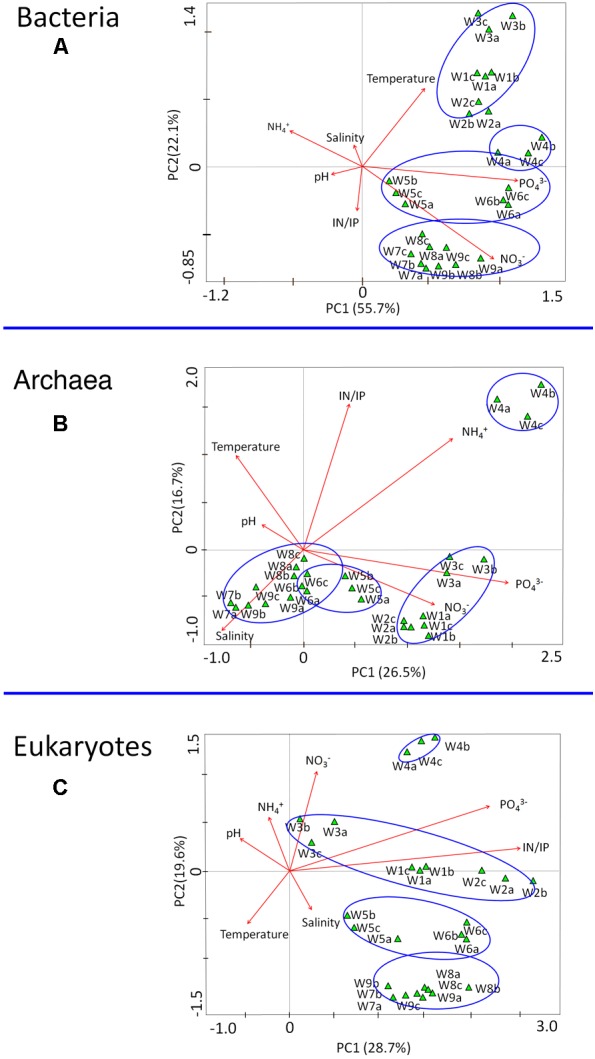
Correspondence canonical analysis (CCA) based on microbial communities (OTUs) and environmental parameters sampled during the bloom. **(A)** Bacteria, **(B)** Archaea, and **(C)** Eukaryotes. W1–W9 refer to the nine different collection time points.

Correspondence canonical analysis (CCA) was used to determine whether correlations in microbial structure were associated with environmental parameters (e.g., temperature, nutrients). Among the chemical factors, PO_4_^3-^ and NO_3_^-^ contributed most to the variance in bacterial communities (**Figure 8A**). In the archaea, NH_4_^+^ and PO_4_^3-^ were the controlling factors associated with population variation (**Figure 8B**), whereas the strongest determinant of community structure in microeukaryotes was the IN/IP ratio (**Figure 8C**). Among the physical parameters, temperature and salinity were related to the composition of bacteria and archaea, respectively. There were no obvious negative or positive correlations between population dynamics and pH value. Additional information about the relationships between genera and environmental parameters as determined by correlation analysis (Pearson correlation coefficient) is shown in **Supplementary Figures S4–S6**.

**FIGURE 9:**
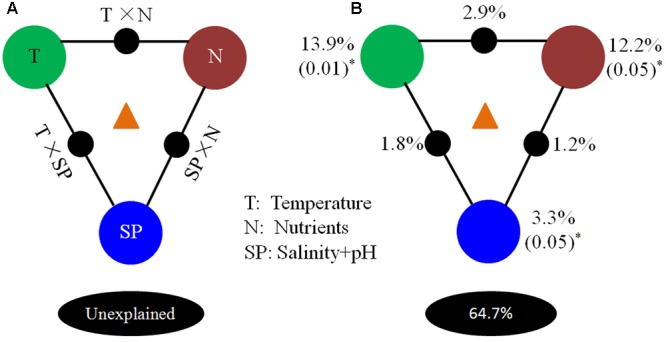
Variation partitioning analysis of microbial distribution explained by environmental factors. **(A)** General outline; **(B)** test environmental parameters, including temperature (T), nutrients (N), and “salinity + pH value” (SP). Each diagram represents the variation partitioned into the relative effects of each factor or combination of factors, in which area is proportional to the respective percentages of variation explained. The edges of the triangles represent the variation explained by each factor alone. The sides of the triangles represent interactions of any two factors, and the middle parts of the triangles represent interactions of all parameters.

A VPA was carried out to partition the contributions of environmental parameters. Results of this analysis showed that temperature (T), nutrients (N), and “salinity + pH” (SP) explained 35.3% of microbial community variation in the OTU data (**Figure 9**), indicating that they were significant factors in determining microbial composition. Independently, T, N, and SP were able to explain 13.9% (*P* = 0.01), 12.2% (*P* = 0.05), and 3.3% (*P* = 0.05) of the total variation observed, respectively. Interactions between T and N, T and SP, and N and SP explained 2.9, 1.8, and 1.2% of variation, respectively. About 64.7% of the microbial community variation in OTU data was not explained by these environmental parameters, indicating that other biotic and/or abiotic factors contribute to these processes.

### Association Network

**FIGURE 10:**
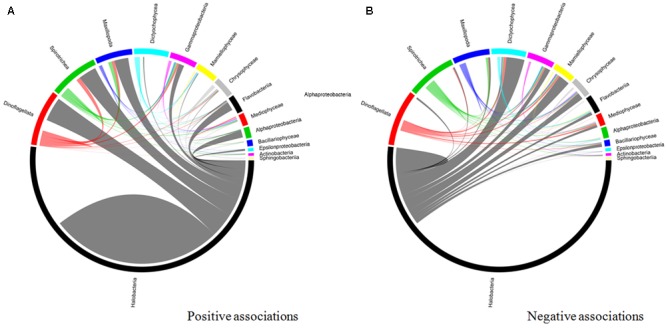
Taxonomic patterns identified within the co-occurrence network. The pop 14 interacting taxon groups are depicted as colored segments in a chord diagram, in which ribbons connecting two segments indicate co-presence **(A)** and exclusion **(B)** links, respectively. Size of the ribbon is proportional to the number of links (co-presence and exclusion) between the OTUs assigned to the respective segments, and color is segment (of the two involved) with the more total links. Links are dominated by *Alphaproteobacteria, Gammaproteobacteria, Flavobacteria*, and *Thermoplasmata.* The chord diagram was generated using the circlized package in R based on the adjacency matrix for the taxon of interest.

An interaction network based on community correlations is shown in **Figure 10**. Among the 15 types of microorganisms analyzed at the class level, there were 14,331 associations, of which 57.6% were positive and 42.4% were negative. These associations represented inter- and intra-phyla interactions in the co-occurring subcommunities, including 663 pairs, 3,521 triplets, and 4,008 quadruplets. Among the positive relationships, all taxa were connected with each other in the network, but *Halobacteria*, dinoflagellates, Spirotrichea, Maxillopoda, and *Gammaproteobacteria* were keystone taxa. In pairwise analyses, these five groups co-occurred most frequently with the other members, particularly with dinoflagellates and *Halobacteria* (**Figure 10A**) (*P* < 0.01). Active interplay was observed in the negative relationships, which appeared to the highest degree in the archaea. The *Halobacteria* was strongly negatively correlated (*P* < 0.01) with several organisms, including Dictyochophyceae, Mamiellophyceae, *Gammaproteobacteria, Flavobacteria*, and some zooplankton (such as Maxillopoda) (**Figure 10B**).

**FIGURE 11:**
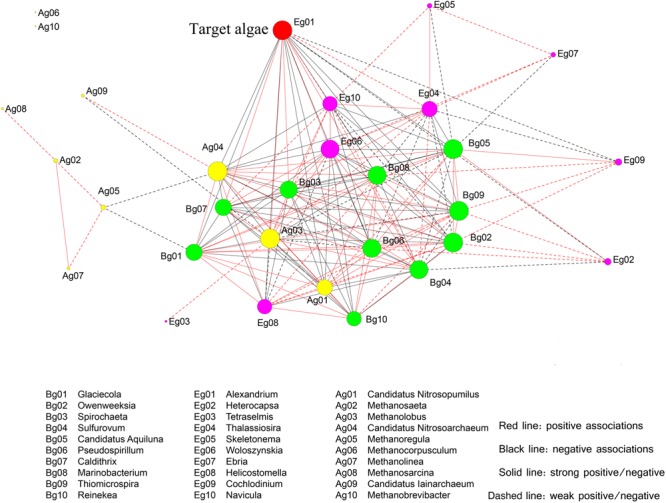
Correlation relationship with the top 30 genera comprising microeukaryotes and target algae (*A. catenella*), based on significant Spearman rank correlation coefficients. The deep red nodes represent target algae (Eg01, the bloom-forming species *A*. *catenella*); green nodes indicate bacterial taxa; yellow nodes indicate archaea; and the light red nodes indicate eukaryotes. Circle size reflects the number of connections the OTUs has with other OTUs. Red bold lines indicate strong positive correlations and red dotted lines indicate positive associations; black bold lines indicate strong negative correlations, black dotted lines indicate negative associations.

To further investigate the correlations between microbial species and the bloom-forming dinoflagellate *A. catenella*, a network was constructed based on significant OTUs (**Figure 11**). For simplicity, we selected the top 30 OTUs, which were analyzed at the genus level. Among the bacterial groups, three members (*Caldithrix, Spirochaeta*, and *Sulfurovum*) had strong positive correlations with *A. catenella*; five members (*Glaciecola, Reineken, Owenweeksia, Candidatus Aquiluna*, and *Thiomicrospira*) were significantly negatively correlated with *A. catenella*; and the remaining species (*Marinobacterium* and *Pseudospirillum*) exhibited weak correlations with the target algae. Within the archaea, three main members (*Methanolobus, Nitrosopumilus*, and *Nitrosoarchaeum*) were correlated with bloom-forming *Alexandrium* sp., whereas other archaea were not significantly associated with algal dynamics. In the microeukaryote biosphere, *Thalassiosira* and *Woloszynskia* directly influenced algal abundance, whereas *Helicostomella* displayed negative effects on algae concentration through grazing or parasitic infection (**Figure 11**). An interesting finding was that three species (*Woloszynakia, Navicula*, and *Methanolobus*) were correlated with both bloom formation and collapse, and exhibited both positive and negative correlations with *A. catenella*.

### Functional Prediction

**FIGURE 12:**
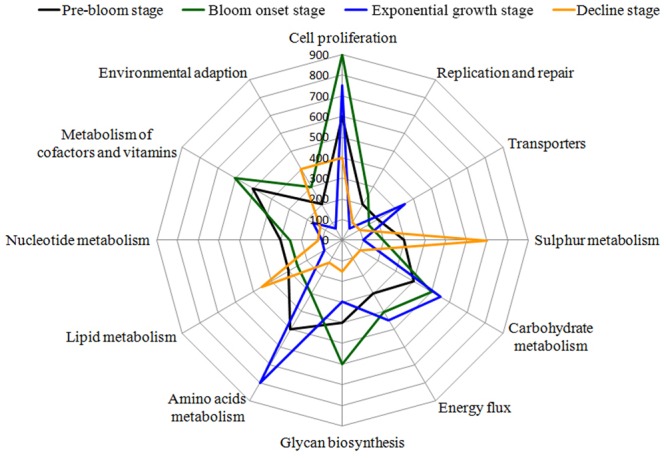
Bacterial taxa associated with algal bloom status are related to several gene functional pathways. Gene functions were predicted from 16S rRNA gene-based microbial compositions using the PICRUSt algorithm to make inferences from KEGG annotated databases. Relative signal intensity was normalized by the number of the genes for each indicated metabolic pathway. The main KEGG pathways, including proteins involved in carbohydrate metabolism, energy flux, lipid metabolism, environmental adaption, cell growth, signal transaction and related physiological behavior are included in the radar graph.

Based on the detection of functional genes, differences in the functional potential of bacterial communities were found among the different bloom stages. The three groups representing major functions (cell proliferation, cofactor and vitamin metabolism, and glycan biosynthesis) showed higher activity in the onset/exponential growth stage [W2–W4] compared with the pre-bloom stage [W1]. Amino acid and carbohydrate metabolism were the main pathways present during the bloom’s peak, leading into decline [W5–W6]. Interestingly, when the bloom began to decline [W7–W9], these metabolic pathways were diminished, whereas sulfur, lipid metabolism, and environmental adaptation genes were enriched during this phase (**Figure 12**).

## Discussion

Ecological studies have suggested the presence of specific algal-microbial interactions based on their co-occurrence in marine systems, which have the potential to influence biogeochemical cycles and shape the community structure of these groups (Amin et al., 2012; Kouzuma and Watanabe, 2015). However, much is yet unknown regarding the mechanisms and dynamics of these interactions, and this remains an area of active research and debate. In this work, we investigated temporal algal and microbial community structure during a bloom of *A. catenella* in an isolated salt pond in the Nauset Estuary on Cape Cod, MA, United States. Self-seeding and annual phytoplankton blooms occur every spring in this system, making Salt Pond an ideal “natural laboratory” for studying algal-microbial dynamics (Crespo et al., 2011; Ralston et al., 2014). Our analysis focused on documenting microbial community structure during algal blooms, and identifying specific associations among bacteria, archaea, and microeukaryotes to provide insights into mechanisms underlying microbial interactions and dynamics.

### Bacterial Community Dynamics

During the earliest bloom stages, the primary bloom-associated bacterial groups were *Gammaproteobacteria, Alphaproteobacteria*, and *Bacteroidetes*. Among these taxa, *Gammaproteobacteria* were the most abundant in OTU sequences. Comprised of predominantly SAR86 and SAR92 clusters, this group is often defined as opportunistic and has a broad generalist substrate spectrum (Skerratt et al., 2002). During the early bloom stages, phytoplankton produce and release carbohydrates, sugar alcohols, and amino/organic acids, carbohydrates, which serve as chemoattractants for these bacterial groups (Buchan et al., 2014). Prior analysis of *in situ* data documented high expression levels of transporters for phosphates, peptides, monosaccharides, and other monomers in these taxa during phytoplankton blooms, indicating that they may be specialized for substrate utilization of algal-derived organic matter (Teeling et al., 2012). In studies of bacterium*–A. catenella* associations the Gulf of Maine (Northwest Atlantic), Hasegawa et al. (2007) found that a dominant and diverse group of *Gammaproteobacteria* was closely associated with *A. catenella*. Our results are similar to this observation and suggest that *Gammaproteobacteria* have a taxon-specific selective capability in the phycosphere niche. In addition, we observed that *Gammaproteobacteria* were still a dominant species during the later bloom stages (**Figure 4**). *Gammaproteobacteria*, and particularly members of the *Alteromonas–Pseudomonas–Vibrio* group, are frequently characterized as r-strategists (Weinbauer et al., 2006), colonizing quickly and promoting growth of more specialized taxa. Hence, *Gammaproteobacteria* provide a certain degree of buffering, which may have promoted stability in the microbial community structure as the *Alexandrium* bloom progressed.

During bloom onset, the bacterial community composition changed, and was dominated by *Bacteroidetes* (especially *Flavobacteria*) and *Alphaproteobacteria* (such as *Roseobacter* and SAR11). *Flavobacteria* are highly diverse, and are capable of degrading high-molecular-weight organics (e.g., cellulose, chitin, and pectin) (Kirchman, 2002). This group is thus regarded as an important component of the microbial loop in coastal phytoplankton blooms (Jones et al., 2010; Williams et al., 2013; Tully et al., 2014). Indeed, Taylor et al. (2014) demonstrated that exopolymer particles (due to algal lysis) area niche occupied by *Flavobacteriales*. Phytoplankton-derived polymeric organic matter is processed by actively hydrolyzing bacteria, as well as a wide variety of opportunistic bacteria that are capable of quenching the products of hydrolysis (Teeling et al., 2012). Prior field studies have reported that certain *Flavobacteria* are abundant during stationary growth and bloom decline (Newton et al., 2011). In this work, *Flavobacteriaceae* were present during all phases of the *A. catenella* bloom and exhibited pronounced fluctuations, perhaps reflecting their role in the processing of organic matter during blooms (Pinhassi et al., 2004). In addition to *Bacteroidetes, Roseobacter* and SAR11 are two other actors in *A. catenella* bloom onset. *Roseobacter* exhibits a high degree of physiological and phenotypic differentiation (Lucena et al., 2012), and has been reported to degrade a variety of dissolved low-molecular-weight organics during phytoplankton blooms, including those associated with HAB species such as *Alexandrium* sp., *Gymnodinium catenatum*, and *Prorocentrum lima* (Lafay et al., 1995; Green et al., 2004; Jasti et al., 2005; Wagner-Dobler and Biebl, 2006). During bloom development, *Roseobacter* is a “pioneer” bacterium, and plays two critical ecological roles in the phycosphere. One is its role as the dominant species in the natural environment, which is achieved through its ability to compete and thrive under low-nutrient conditions (Pinhassi and Berman, 2003), and the second is its provision of sulfur compounds that stimulate algal growth (Tan et al., 2015). These roles reflect both ecological strategies and contributions of this dominant group. The SAR11 (such as *Rhizobiales*) is another active producer of S and N during blooms (Teeling et al., 2012). Shifts in OTU numbers we observed corroborates the hypothesis that members of the *Rhizobiales* clade are capable of rapidly changing metabolic functioning in response to changing conditions during phytoplankton blooms, thus enhancing their competitive ability (Gao, 2015).

When an algal bloom begins to decline, bacterial community structure becomes more diverse and complex. Phytoplankton release macromolecules and small molecules (e.g., polysaccharides, proteins, lipids, and other material from cell lysis) in response to nutrient-limiting conditions, which further stimulates heterotrophic bacterial activity (Buchan et al., 2014). Indeed, this phenomenon was observed in the current study, exemplified by the substantial increase in *Actinobacteria* and *Cyanobacteria* (**Supplementary Figure S3A**). In the oceanic environment, *Actinobacteria* helps to decompose organic matter (e.g., dead algae) for uptake by phytoplankton. Several researchers have reported that *Actinobacteria* are closely linked to the diatom/dinoflagellate blooms (Eckert et al., 2012; Bunse et al., 2016), which could help to explain their wide distribution and high abundances following blooms. *Cyanobacteria* are fast-growing nutrient opportunists in the phycosphere environment, and can compete with other slow-growing nutrient specialists via exploitative resource competition (Vallina et al., 2014). This is largely governed by substrate availability, as algal-derived substrates provide ecological niches for specific populations and concurrently generate a selective advantage for bacteria that are fundamentally opportunistic (Taylor et al., 2014). In addition, photosynthetic cyanobacterial taxa such as *Synechococcus, Chroococcales*, and *Oscillatoriales* were detected in samples collected during bloom decline and contributed about 10–30% to overall abundance (data not shown), which is in line with previous reports suggesting a role for *Cyanobacteria* in sinking particle flux (as decomposers of organic material from dead algae) (Palenik, 2015).

It is important to note that some bacteria exhibit dual roles during different stages of the bloom, such as *Cytophaga–Flavobacterium–Bacteroides* (CFB) and *Rhodobacterales*. In this work, the most marked fluctuations were found in heterotrophic taxa, including the CFB members, which may reflect their ability to quickly establish and grow under changing conditions (algal lysis, grazing, and nutrient depletion) during the bloom. Previously, Rinta-Kanto et al. (2012) demonstrated that heterotrophic microbes decrease energy conservation in response to blooms, effectively shifting bioreactive compounds assimilated from the DOM (dissolved organic matter) pool, and altering the surfaces of cells in a manner that effectively promotes both adhesion and particle formation. This is a survival strategy in response to increasing competition for resources. In addition, CFB are also the most common algicidal bacteria (Kodama et al., 2006), which may also explain why CFB are found during the decay of the blooms (Zhou et al., 2016a). Another widespread and commonly found group is *Rhodobacterales*, which comprise large fractions of the total bacterioplankton. Given its antagonism toward other bacteria, this group may be an important regulator of HAB dynamics (Roth P. A. et al., 2008; Roth P. B. et al., 2008; Li et al., 2013). Our results showed that *Rhodobacterales* can wax and wane with *Alexandrium* sp., thus exhibiting non-equilibrium coexistence.

### Archaeal Community Structure

Archaea have been largely ignored as potential agents in phytoplankton dynamics, although a few studies indicated a euryarchaeal association with eukaryotic phytoplankton (Needham and Fuhrman, 2016). In this work, we found that most archaeal members belonged to Marine Group II (**Figure 5**), and their communities during the bloom were less complex compared with bacterioplankton. Changes in archaeal community composition during blooms have been sporadically reported in blooms of diatoms (Kim et al., 2014), cyanobacteria (Xing et al., 2012), and dinoflagellates (such as *Scrippsiella trochoidea*) (Lumarie, 2006; Tan et al., 2015); and most demonstrated that methanogens were predominant members of the archaeal biosphere. Similar to the aforementioned results, we also found that the dominant group throughout the bloom was comprised of methanogenic archaea (nearly 50%) (**Figure 5**). During the pre-bloom period, *Methanomicrobiales* and *Methanocellales* were the predominant genera. The *Methanocellales* are phylogenetically diverse, and may play a significant role in ecosystem functioning through their ability to process complex compounds into simpler molecules in both anaerobic and aerobic habitats (Chouari et al., 2015). This ability to convert polysaccharides into compounds that are usable by other taxa might allow *Methanocellales* to be more competitive than groups reliant on other energy sources. Our observations suggest that the occurrence of *Methanocellales* at the beginning of the bloom may have been associated with the less eutrophic conditions present at that time.

Two additional methane-metabolizing members, *Methanosarcinales* and *Methanobacteriales*, co-occurred in the mid- and post-bloom stages, potentially as syntrophic partners inorganic biomass degradation (Chauhan et al., 2006). In addition, it should be noted that different algal hosts may have been associated with different methanogenic archaea. In diatoms, *Methanomicrobiales* constituted the resident population due to its fast growth response to nitrate and phosphate availability (den Haan et al., 2013), whereas in our study, *Methanobacteriales* and *Methanomicrobiales* were both commonly associated with *Alexandrium* sp. This may reflect their growth responses to the variety of organic compounds produced by different phytoplankton host, which selects for different archaeal communities (Schwarz et al., 2008).

During the post-bloom stage, AOA emerged, and *Nitrosopumilaceae* was particularly abundant. AOA activity provides an efficient way of removing ammonium waste secreted by the host and prevents toxic NH_4_^+^ accumulation, thus strengthening the relationship between phytoplankton and their symbionts. Pearman et al. (2016) further demonstrated that this recycling of ammonia is the predominant driving force in AOA community composition. One additional observation of note pertains to the *Halobacteriales*, which were enriched during the bloom’s terminal phase. The AOA archaeal communities present in Salt Pond late in the bloom were consistently associated with *A. catenella*, suggesting that these symbionts may play a key role in NH_4_^+^ detoxification for their host, and thus could significantly impact the nitrogen cycle in the phycosphere. One additional observation of note pertains to the *Halobacteriales*, which were enriched during the bloom’s terminal phase. Certain taxa in this group are capable of growing both aerobically and anaerobically, and they have a novel means of synthesizing energy through photosynthesis. In addition, *Halobacteriales* actively degrades organic matter in salty environments. The higher abundance of *Halobacteria* may thus indicate involvement in organic matter degradation, thus supplying inorganic compounds for algae (Zhou et al., 2014). This could be beneficial to resident algae since dissolved organic matter and nutrients concentrations are typically low during the pre-bloom stage.

### Microeukaryote Community Structure

In the phytoplankton biosphere, obvious variations in community structure associated with bloom dynamics were observed. Significant differences in the Shannon– and Simpson indices were found among the different HAB stages (**Figure 2**), which indicated a lower level of α-diversity in whole bloom period. A significant shift in the phytoplankton community structure was also reflected in β-diversity measures (**Figure 3**). Pre-bloom phytoplankton communities were characterized by high diversity, which included both diatom and dinoflagellate taxa. Upon bloom initiation, diatom abundance declined markedly. This suggests that diatom growth may have been negatively influenced by the high abundance of *Alexandrium* sp. Allelopathic and auto-inhibition effects play important roles in competition between algae, and may have contributed to the dynamics we observed between these groups (Yamasaki et al., 2007; Wang et al., 2013).

During the early stages of the bloom, communities were dominated by Dinophyceae (*Alexandrium, Heterocapsa, Woloszynskia, Cochlodinium*, and *Polykrikos*), with various green algae or diatoms (*Tetraselmis, Micromonas, Bathycoccus, Thalassiosira, Skeletonema*, and *Navicula*) as minor contributors (**Figure 6**). Several days later, the number of algal cells peaked, and the eukaryotic community was comprised almost exclusively of *Alexandrium* sp. (90%). Subsequent to the bloom’s peak, multiple phytoplankton groups co-occurred, ranging from nutrient specialists to nutrient opportunists (such as green algae). During bloom decline, diatoms and green algae comprised nearly 50% of eukaryotic OTUs. Algal activities led to rapid exhaustion of nutrients, and in conjunction with other taxa (e.g., diatoms, eukaryote grazers), contributed to bloom termination. Other unmeasured hydrochemical characteristics and/or factors not examined in this study likely contributed as well. Algal mortality during this stage dramatically increased the substrate available to other microeukaryotic communities, and the most obvious shift in community composition occurred during bloom termination.

Other non-abundant *Prymnesiophyceae* species, haptophytes, comprised < 3% of the eukaryotic community and only appeared during the post-bloom stage. Despite high levels of haptophytes reported during blooms of other dinoflagellates (Friocourt et al., 2012), comparatively low haptophyte OTUs were observed in the dataset collected during this *Alexandrium* bloom. Several reasons for this finding are possible: (1) different hosts have different associated microeukaryotic communities; (2) higher copy numbers of ciliate and diatom 18S rRNA gene sequences dwarfed haptophyte DNA; and (3) primer mismatches or difficulties amplifying GC-rich haptophyte DNA, which resulted in low haptophyte tag yield. Despite the relatively small OTU tag numbers in our dataset, haptophytes are known to be opportunistic, and utilize urea and phosphonate as alternative sources of N and P (Pearman et al., 2016). In this study, a negative correlation was observed between haptophytes and *Alexandrium* (**Figure 6**), which may indicate that these species have the ability to compete with algae for nutrients and thus contribute to *Alexandrium* bloom termination.

Among the zooplankton communities, the most abundant taxa were protists (*Apicomplexa*), Arthropoda (*Maxillopoda*), ciliate (*Ciliophora*), and rotifera. Their abundance was enriched during the bloom termination and post-bloom stages. Most of these taxa are phytoplankton grazers, and are the primary drivers in top–down control that can play a critical role in bloom decline. Our results are similar to those reported by Alacid et al. (2015), i.e., that protists (such as the dinoflagellate parasitoid *Parvilucifera sinerae*) may be an important factor in the termination of *Alexandrium minutum* blooms in the natural environment. Regarding the observed increase in ciliates during the post-stage, one possible reason is the availability of easily digestible taxa such as green algae and diatoms, both of which were dominant at the end of the bloom (**Figure 6**). Fungi were another cornerstone taxa during the transition from a phytoplankton dominated community to non-phytoplankton-dominated community. In this work, *Basidiomycota* and *Ascomycota* were the primary members detected, and their abundance was increased during bloom termination. These results are similar to our previous observations (Sun et al., 2017), and suggest that there might be a saprophytic association between fungi and the decomposition of *A. catenella* biomass.

In addition to bacteria, archaea, and microeukaryotes, viruses are another important regulator of algal bloom activity. Tomaru et al. (2007) reported that viral infection by HcRNAV significantly impacts the population dynamics of *H. circularisquama*, both in terms of population abundance (biomass) and structure (clonal composition). Recent work further indicates that viruses influence algal host physiology and behavior (Park et al., 2004; Cai et al., 2011). The present work did not carry out an analysis of viral impacts; however, more detailed knowledge is clearly needed regarding the interactions between viruses and their algal hosts, and the ecological implications for algal bloom events.

### Environmental Factors Regulating Microbial Structure

Among the environmental parameters analyzed, we found that temperature was strongly correlated with bacterial diversity, and also influenced archaeal communities (**Figures 8A,B**). These results indicate that temperature was the major abiotic force shaping microbial community structure in Salt Pond, which is consistent with past studies that documented a pronounced impact of temperature on plankton composition (Niu et al., 2011; Hampton et al., 2014; Frenken et al., 2016). For example, in the German Bight Lucas et al. (2015) identified temperature as a major niche-defining factor that indirectly influenced patterns of short-term bacterioplankton succession during phytoplankton blooms. Together, these findings indicate that temperature-mediated succession of the plankton community drives variation in microbial community composition (Pomeroy and Wiebe, 2001). In the present study, salinity was also identified as a primary physical factor driving 16S diversity, particularly archaeal community composition (**Figure 8B**), which may exhibit sensitivity to osmotic pressure. Salinity was previously shown to be a principal driving force of archaeal community patterns at the global scale (Auguet et al., 2010), these results demonstrate the importance of salinity in determining archaeal community structure at local scales. It is worth noting that although other environmental factors measured in this study were not associated with community structure, it is certainly possible that other unmeasured water chemistry parameters participated in community dynamics. Similar to previous findings (Bolhuis and Stal, 2011), salinity was related to the archaeal dynamics in Salt Pond, but is only one of the factors determining archaeal community structure. Future studies into the influence of salinity on these communities, including the effect of different ionic compounds and the buffering capacity of seawater, would be helpful in clarifying the relevance of salinity to archaeal populations.

In addition to physical factors, N and P are two key chemical elements that influence microbial communities through their effects on substance utilization and growth (Garcia et al., 2016). The acquisition and efficiency of N/P are linked to specific nutrient stoichiometry, thus determining the succession of bloom-forming phytoplankton taxa such as *Alexandrium* sp. (Tsuchiya et al., 2014). In this work, we observed an inverse relationship between phytoplankton biomass and nutrient concentrations during the bloom. Following bloom initiation, concentrations of NO_3_^-^ and NH_4_^+^ decreased during bloom development (**Figures 1B,C**), presumably due to uptake and utilization by *Alexandrium* sp. (Ji et al., 2018), and increased thereafter as the bloom declined. During this same time period, P concentrations also decreased at the bloom’s peak stage. Phytoplankton communities may thus have experienced P-limitation due to its depletion during the exponential growth phase (Ji et al., 2018). Microbial biomass was highest following the bloom’s peak, when nutrient levels again rose. These results support prior findings in which algal bloom community succession was regulated by nutrient availability, which contributes to bottom–up control over both phytoplankton and microbial populations (Teeling et al., 2012).

The environmental data were then analyzed to determine which parameters drive community variability. These analyses included temperature, nutrients, and hydrochemical parameters (pH and salinity) as explanatory variables. These factors independently explained 13.9, 12.2, and 3.3% of the total observed variations, respectively (**Figure 9**). It partly supports the principle of “everything is everywhere, but the environment selects” (O’Malley, 2008). Abiotic (physical conditions and nutrient availability) and biotic factors (co-occurrence or exclusion) have long been regarded as the primary drivers shaping the distribution of microbial communities during algal events; the former have been considered to have a stronger effect (Rohwer and Thurber, 2009; Smetacek, 2012; Worden et al., 2015). However, Lima-Mendez et al. (2015) reported that environmental factors contributed to just 18% of community variation of microbes at the global ocean scale. Similarly, in this study, just 35.3% of microbial community variation in the OTU data was explained by above-mentioned parameters, indicating that other abiotic factors not sampled (such as DOC, DOM, or ROS abundance) or biotic parameters (e.g., uncovered community interactions) may be important drivers of community structure. These findings, however, may also indicate that the effect of abiotic factors on community structure is more limited than was previously assumed (Worden et al., 2015). Further investigation of the influence of physical-chemical parameters, particularly over longer time scales, is needed to better characterize the relative contribution of these factors on community variation.

### Association Network Correlations

Network analysis has been shown to be an effective tool for characterizing biotic interactions and associations at various taxonomic levels (Lupatini et al., 2014). In this study, we observed a variety of different co-occurrence patterns among dominating prokaryotes and eukaryotes. In bacterial communities, diverse inter- and intra-relationships were observed at the class level (**Figures 10A,B**). Furthermore, the putative keystone nodes in the network were highly abundant (e.g., Spirotrichea, Maxillopoda, and *Gammaproteobacteria*), suggesting that these groups are important in maintaining network structures in the microbial community. Several examples of interplay (positive or negative) among these groups have been reported previously (Chaffron et al., 2010), which support the biological relevance of the findings reported here. In the archaea, more negative than positive interactions were observed in *Halobacteria*, suggesting that competition may occur more frequently than cooperation between archaea and other bacterioplankton. Notably, Zheng et al. (2013) showed that patterns of diversity in archaea are primarily driven by stochastic processes; our results lend support to this view and suggest that competition contributed to the random process of archaeal distribution. In the eukaryotes, relatively low connectivity (focusing on dinoflagellates) was observed in the network, indicating that eukaryotes were less affected by surrounding microorganisms and thus exhibited higher stability (Zhou et al., 2010). Overall, our results suggest that community composition was shaped by both competitive and cooperative forces. Although the exact contribution of biotic interactions with community variations is not presently known, this work provides possible clues to foster a better understanding regarding which keystone taxa may be present in the microbial communities during blooms, how they interact, and which bioprocesses they influence in the phycosphere environment.

A multiplicity of inter- and intra-specific associations (direct or indirect) within the network were further analyzed in order to illuminate correlations between the primary microbes and the bloom-forming algae *Alexandrium* sp. (**Figure 11**). Among the bacterial groups, positive correlations were observed during the bloom that suggested certain functional interdependencies between bacteria and algae. In this study, *Alexandrium* sp. was positively correlated with *Caldithrix, Spirochaeta*, and *Sulfurovum*. These keystone species are characterized as opportunistic and play multiple roles, including the degradation of organic matter (Seyedsayamdost et al., 2011), metabolizing dimethylsulfoniopropionate (DMSP) (Moran et al., 2012). And essentially serving as a nutrient “cooker” (producing vitamins, iron, and dissolved organic material) (Jones et al., 2010). These positive correlations indicated that these species would have been adapted to the surrounding environment, and may have played a role in algal bloom duration (Tan et al., 2015). In contrast, certain negative correlations between bacteria (such as *Glaciecola, Reineken*, and *Owenweeksia*) and *Alexandrium* sp. were also observed, reflecting the appearance of nutrient competitors or algicidal bacteria during the blooms. The former are more efficient than algae at acquiring P and N from organic compounds (Løvdal et al., 2008), while the latter can release molecules that kill or lyse algal cells (Mu et al., 2007; Amin et al., 2012).

Similar to bacteria, certain archaeal species were directly or indirectly correlated with *Alexandrium* sp. dynamics. *Methanolobus* was the primary group present during bloom formation, whereas *Nitrosopumilus* and *Nitrosoarchaeum* were the key members during bloom decline and termination. As a member of the methanogenic archaea, *Methanolobus* exhibit positive effects as symbionts, due in part to their participation in the N cycle in the phycosphere environment (Needham and Fuhrman, 2016). *Nitrosopumilus* and *Nitrosoarchaeum* likely were the major N-source competitors with algae because of their ability to utilize and oxidize ammonia (Metcalf et al., 2012). In addition to the aforementioned species, certain archaea exhibited strong interdependence with other taxa. These “hub species” (highly linked species within their own module) may function to promote the stability and maintenance of the network. In the microeukaryotic biosphere, positive correlations with *Alexandrium* sp. were observed during the algal bloom period, including *Thalassiosira* and *Woloszynskia*, but negative correlations were more frequent. *Helicostomella* exhibited the most significantly negative correlations with *Alexandrium* sp., indicating that competitors are primary drivers in the top–down process. An additional and interesting finding was that three genera (*Woloszynakia, Navicula*, and *Methanolobus*) had a dual correlation with bloom growth and collapse, suggesting the ability to adapt the changing environmental conditions associated during the algal bloom, based on their documented genotypic and metabolic diversity (Cram, 2015). It should be noted that the correlations between microorganisms and *Alexandrium* sp. reported in this study are based on relative abundance; as improved quantification methods become available to researchers, it will be possible to investigate these relationships using a more statistically robust approach.

### Potential Microbial Function

To further investigate the ecological role of phycosphere microbiota, functional predictions were analyzed using the PICRUSt algorithm (Langille et al., 2013). To reduce complexity, only bacteria were analyzed. From the functional modules, we found that genes related to cell proliferation, carbohydrate metabolism, and energy flux were highly abundant during bloom onset, whereas functional modules involved in lipid, sulfur metabolism, and environmental adaption were over-represented during the bloom decline stages (**Figure 12**). Using Geochip, Yang et al. (2016) found that carbon degradation activity significant increased during a bloom of *Akashiwo sanguinea*, which was consistent with our results. Energy metabolism was maintained at relatively high levels during the onset and exponential stages, which may have contributed to the *Alexandrium* bloom’s duration. Similar results were observed in *A. sanguinea*, and through promotion of nitrogen (ammonium) accumulation, organic acid utilization, and carbon assimilation (Kudela et al., 2008; Rinta-Kanto et al., 2012). In this study, genes related to lipid and sulfur metabolism were mainly enriched in the onset and decline phases, which indicate that S and P availability were important for both bloom initiation and termination. Additionally, modules for amino acids metabolism and active transporters were over-represented in log-phase and during the beginning stage of bloom termination. These results show that bacterial functions affect phycosphere metabolic activity and algal bloom development trajectories.

## Conclusion

This work analyzed microbial (bacteria, archaea, and microeukaryotes) assemblages from the natural environment during a phytoplankton bloom event. The microbial response to algal blooms exhibited high heterogeneity and complex dynamics, which comprised the succession of distinct populations exhibiting distinct functional profiles. Further, the association networks showed that algal and microbial community structure observed during the bloom was shaped by environmental factors as well as biotic interactions. The results highlight the importance of microbial network interactions as a robust tool to help understand the fate of HABs. It should be noted that we could not investigate the metaproteomes or metabolomes of phycosphere microorganisms in the present study. Future research using integrated approaches, such as flow cytometry and “Omics” methods, will continue to improve our understanding of microbial community interactions during HAB events, and could be used to further explore causal relationships between microbial diversity and activity, and algal blooms.

## Author Contributions

JZ and ZC performed the experiments. JZ and MR drafted the manuscript. TS and DK collected the samples and analyzed the data. JZ and MR prepared the figures and tables. DA, ZC, and DK completed critical comments and revision.

## Conflict of Interest Statement

The authors declare that the research was conducted in the absence of any commercial or financial relationships that could be construed as a potential conflict of interest.

## Footnotes

1 http://cmore.soest.hawaii.edu

2 http://drive5.com/uparse/

3 https://cran.r-project.org/web/packages/circlize/index.html

4 http://www.phylotemp.microeco.org

## Supplementary Material

FIGURE S1 Morphology of *Alexandrium catenella* under light microscopy.

FIGURE S2 Photographs of DAPI-strained filter samples under fluorescence microscopy (1000×). 1 mL of fixed sample was filtered onto 0.22 μm pore-size polycarbonate filters. The blue spots show bacteria particles in the sample (circles show the smaller bacteria). The scale bar is 10 μm.

FIGURE S3 Relative abundance of bacteria at phylum level **(A)**, and *Proteobacteria* at class level **(B)**.

FIGURE S4 Correlation analysis between relative abundances of the 100 most abundant bacterial OTUs and environmental variables based on Pearson correlations. Correlation values depict *r*-values of Pearson correlations. Statistical significance levels: ^∗^*P* < 0.05, ^∗∗^*P* < 0.01, and ^∗∗∗^*P* < 0.001.

FIGURE S5 Correlation analysis between relative abundances of the most abundant archaeal OTUs and environmental variables based on Pearson correlations. Correlation values depict *r*-values of Pearson correlations. Statistical significance levels: ^∗^*P* < 0.05, ^∗∗^*P* < 0.01, and ^∗∗∗^*P* < 0.001.

FIGURE S6 Correlation analysis between relative abundances of the 100 most abundant microeukaryote OTUs and environmental variables based on Pearson correlations. Correlation values depict *r*-values of Pearson correlations. Statistical significance levels: ^∗^*P* < 0.05, ^∗∗^*P* < 0.01, and ^∗∗∗^*P* < 0.001.
