# Supplementary figures and images for: Microbial Community Structure and Associations During a Marine Dinoflagellate Bloom
Source: Front Microbiol. 2018 Jun 6;9:1201. doi: 10.3389/fmicb.2018.01201 (PMC5998739; doi:10.3389/fmicb.2018.01201)

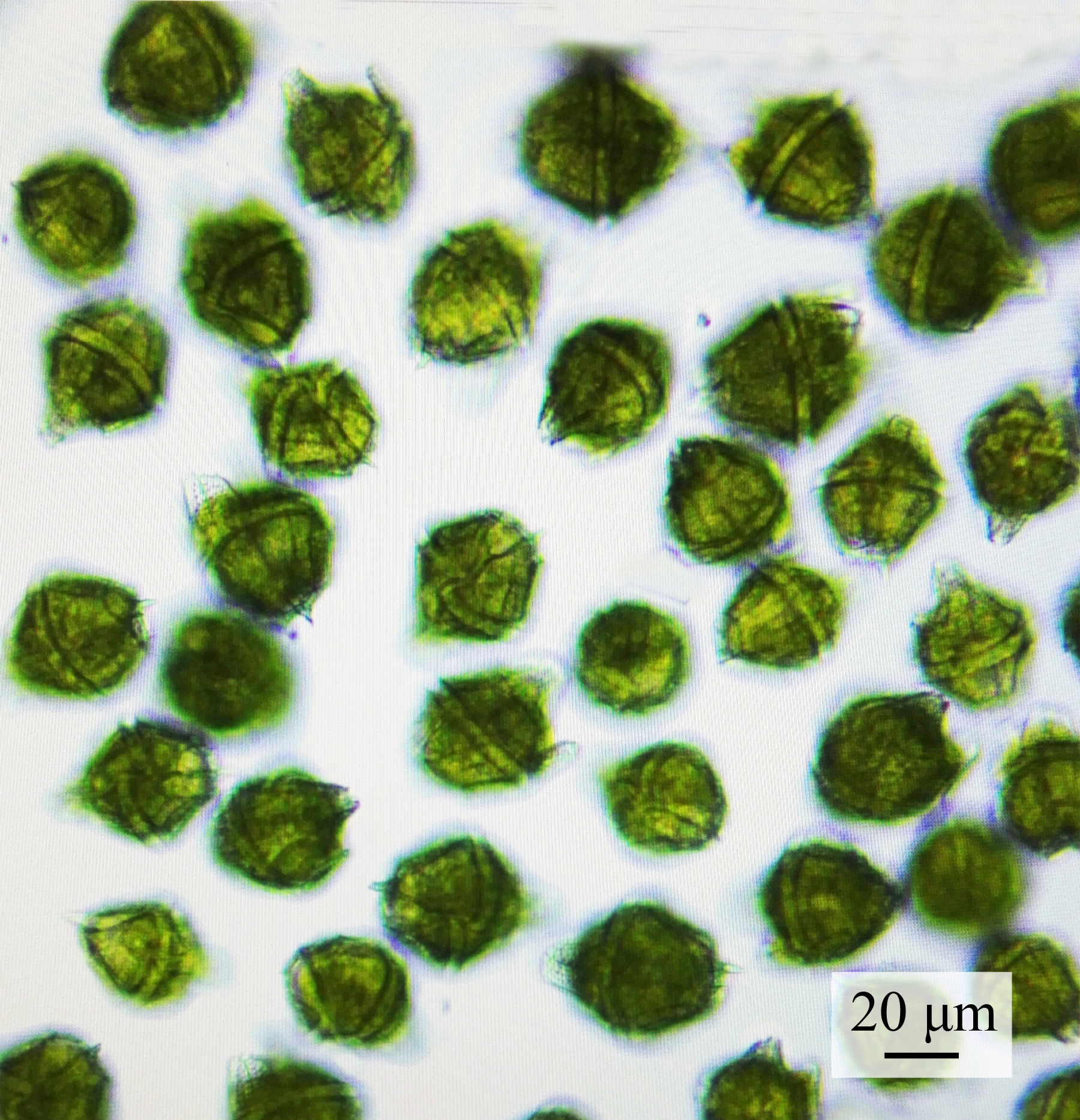

Supplement: FIGURE S1 — Morphology of Alexandrium catenella under light microscopy. [file Image_1.JPEG]

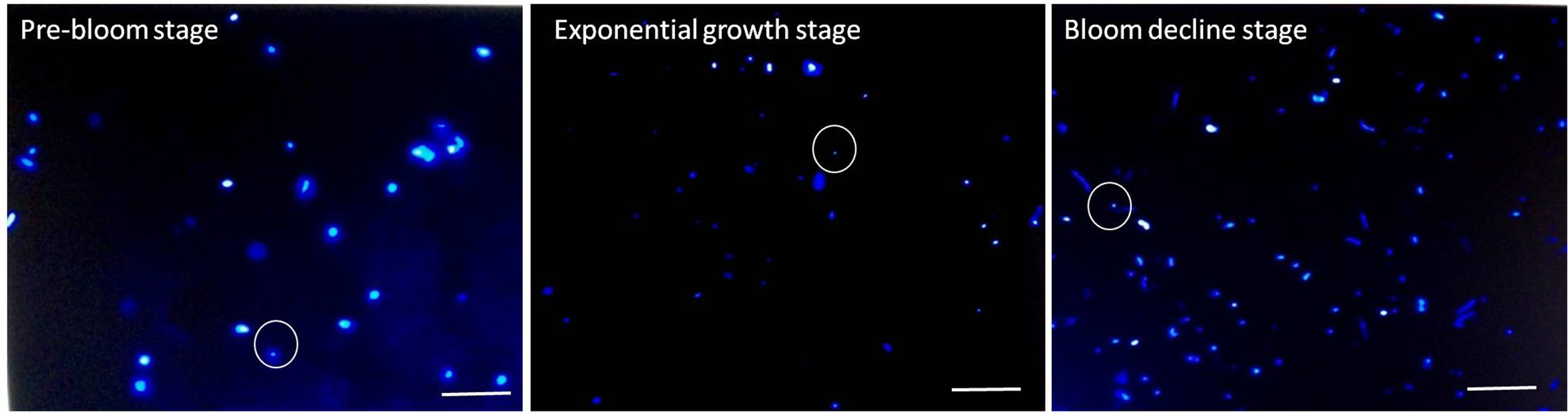

Supplement: FIGURE S2 — Photographs of DAPI-strained filter samples under fluorescence microscopy (1000×). 1 mL of fixed sample was filtered onto 0.22 μm pore-size polycarbonate filters. The blue spots show bacteria particles in the sample (circles show the smaller bacteria). The scale bar is 10 μm. [file Image_2.JPEG]

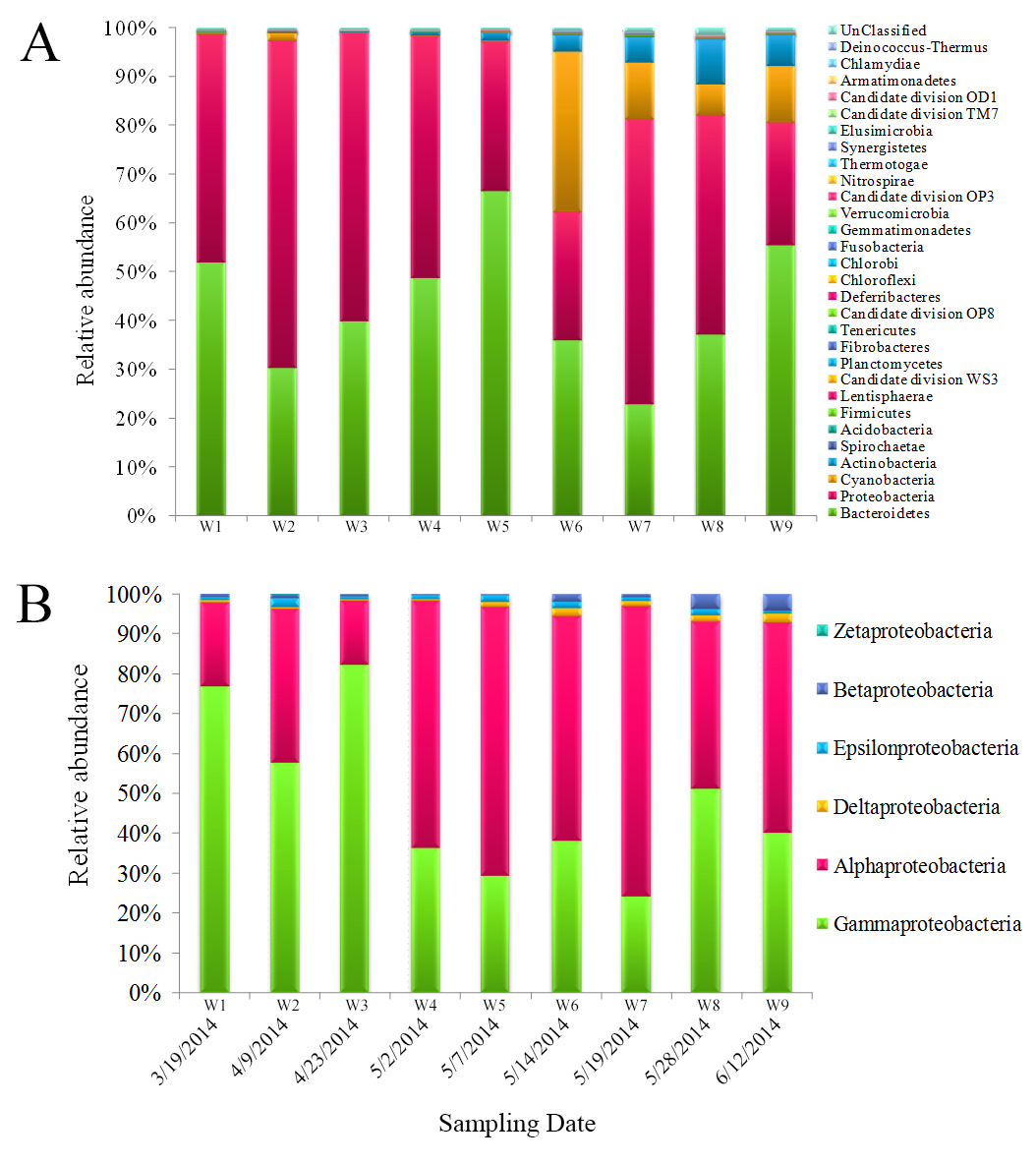

Supplement: FIGURE S3 — Relative abundance of bacteria at phylum level (A), and Proteobacteria at class level (B). [file Image_3.JPEG]

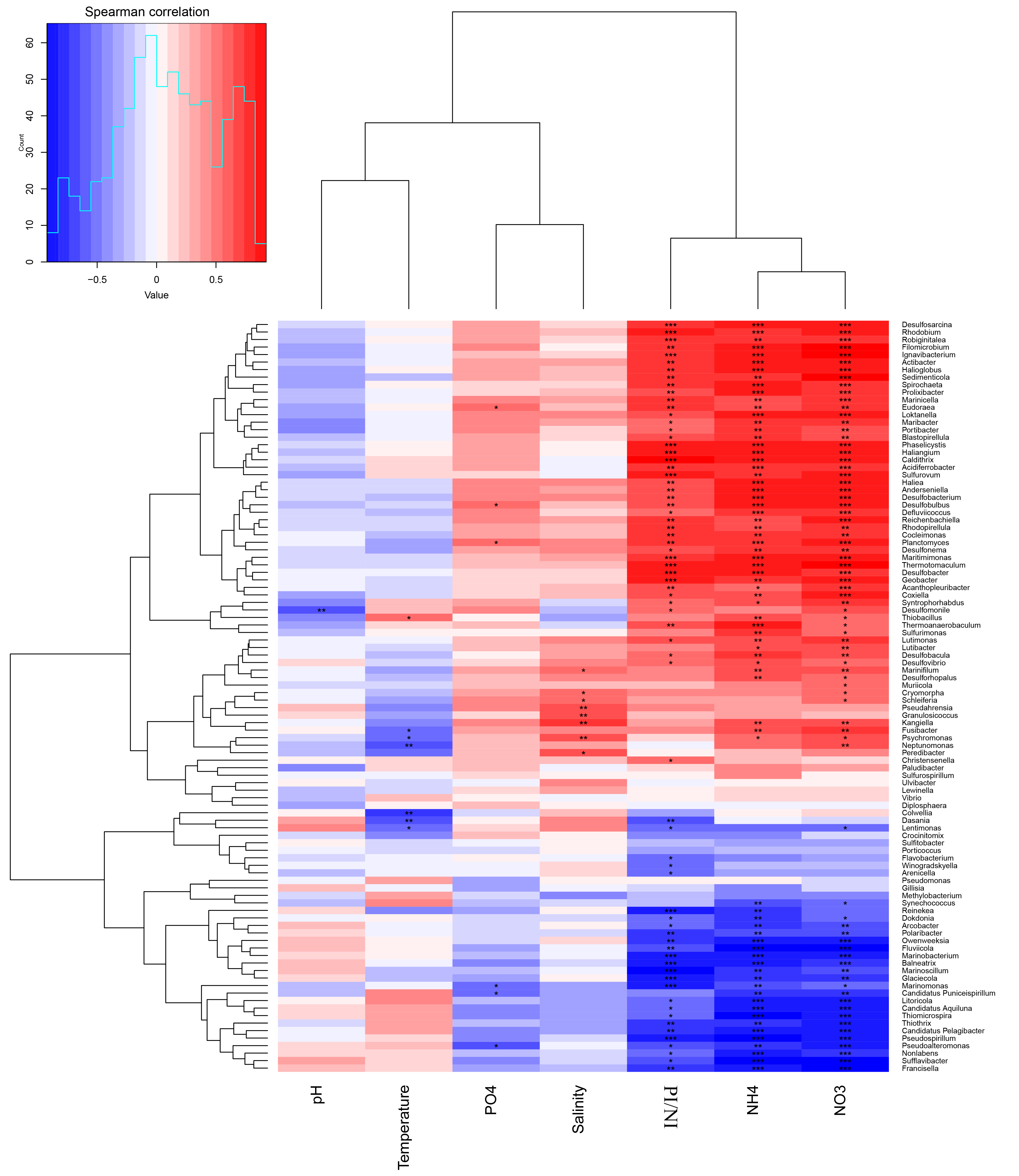

Supplement: FIGURE S4 — Correlation analysis between relative abundances of the 100 most abundant bacterial OTUs and environmental variables based on Pearson correlations. Correlation values depict r-values of Pearson correlations. Statistical significance levels: ∗P < 0.05, ∗∗P < 0.01, and ∗∗∗P < 0.001. [file Image_4.JPEG]

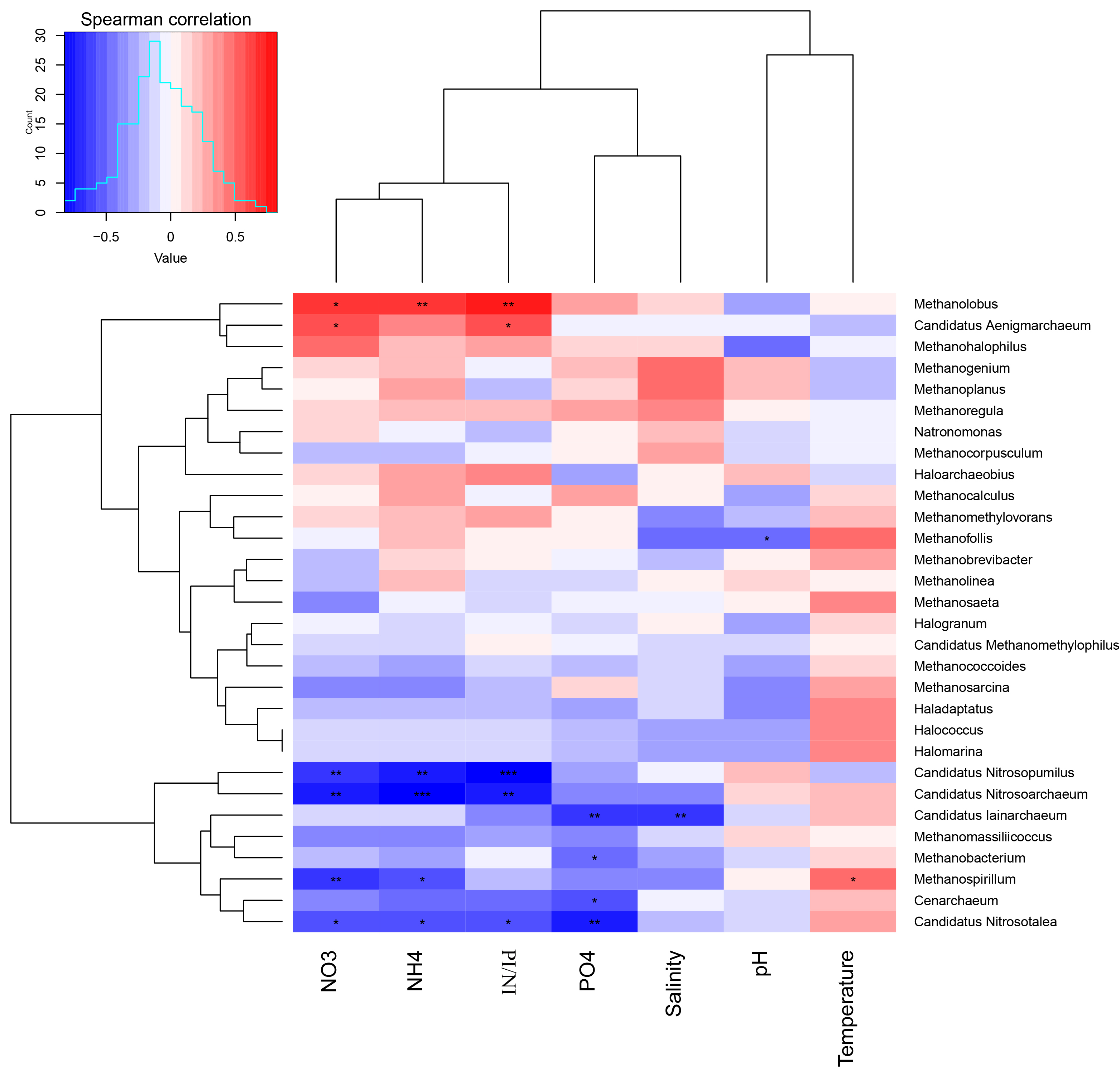

Supplement: FIGURE S5 — Correlation analysis between relative abundances of the most abundant archaeal OTUs and environmental variables based on Pearson correlations. Correlation values depict r-values of Pearson correlations. Statistical significance levels: ∗P < 0.05, ∗∗P < 0.01, and ∗∗∗P < 0.001. [file Image_5.JPEG]

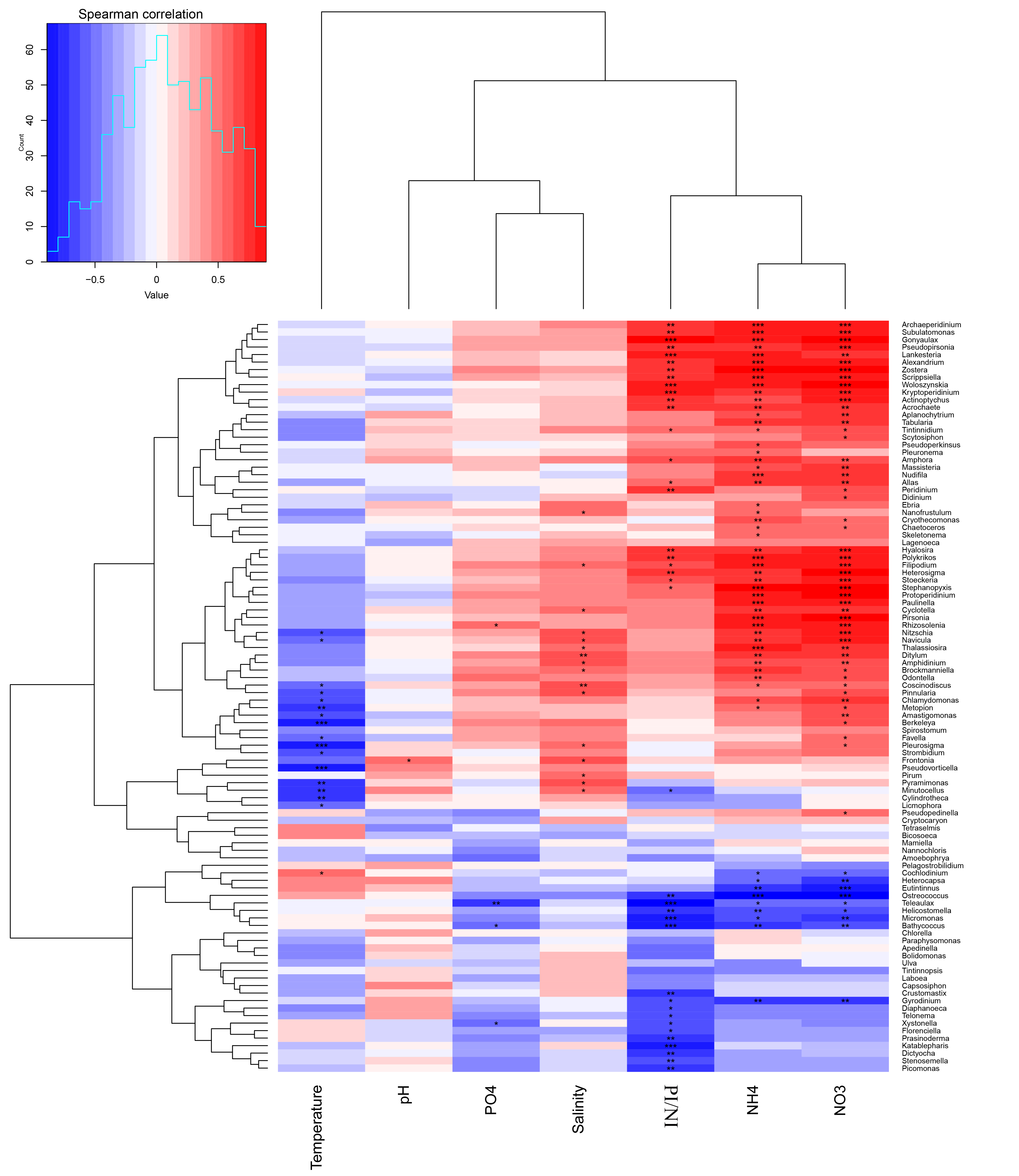

Supplement: FIGURE S6 — Correlation analysis between relative abundances of the 100 most abundant microeukaryote OTUs and environmental variables based on Pearson correlations. Correlation values depict r-values of Pearson correlations. Statistical significance levels: ∗P < 0.05, ∗∗P < 0.01, and ∗∗∗P < 0.001. [file Image_6.JPEG]
